# Novel mutations and phenotypic associations identified through *APC*, *MUTYH*, *NTHL1*, *POLD1*, *POLE* gene analysis in Indian Familial Adenomatous Polyposis cohort

**DOI:** 10.1038/s41598-017-02319-6

**Published:** 2017-05-22

**Authors:** Nikhat Khan, Anuja Lipsa, Gautham Arunachal, Mukta Ramadwar, Rajiv Sarin

**Affiliations:** 1 Sarin Lab, Advanced Centre for Treatment, Research and Education in Cancer (ACTREC)-Tata Memorial Centre, Navi Mumbai, India; 2 Homi Bhabha National Institute, Training School Complex, Anushakti Nagar, Mumbai, 400085 India; 3 Clinical Genetics Unit, Christian Medical College and Hospital, Vellore, India; 4 Department of Pathology, Tata Memorial Hospital-Tata Memorial Centre, Mumbai, India

## Abstract

Colo-Rectal Cancer is a common cancer worldwide with 5–10% cases being hereditary. Familial Adenomatous Polyposis (FAP) syndrome is due to germline mutations in the *APC* or rarely *MUTYH* gene. *NTHL1*, *POLD1*, *POLE* have been recently reported in previously unexplained FAP cases. Unlike the Caucasian population, FAP phenotype and its genotypic associations have not been widely studied in several geoethnic groups. We report the first FAP cohort from South Asia and the only non-Caucasian cohort with comprehensive analysis of *APC*, *MUTYH*, *NTHL1*, *POLD1*, *POLE* genes. In this cohort of 112 individuals from 53 FAP families, we detected germline *APC* mutations in 60 individuals (45 families) and biallelic *MUTYH* mutations in 4 individuals (2 families). No *NTHL1*, *POLD1*, *POLE* mutations were identified. Fifteen novel *APC* mutations and a new Indian *APC* mutational hotspot at codon 935 were identified. Eight very rare FAP phenotype or phenotypes rarely associated with mutations outside specific *APC* regions were observed. *APC* genotype-phenotype association studies in different geo-ethnic groups can enrich the existing knowledge about phenotypic consequences of distinct *APC* mutations and guide counseling and risk management in different populations. A stepwise cost-effective mutation screening approach is proposed for genetic testing of south Asian FAP patients.

## Introduction

Inherited predisposition is seen in 5–10% of all colorectal cancers (CRC). Major forms of hereditary colorectal cancer include the non-polyposis Lynch syndrome and the Familial Adenomatous Polyposis (FAP) syndrome^1^. Colorectal polyposis syndromes are characterized by multiple adenomatous or hamartomatous polyps and account for about 1% of all CRC cases. The adenomatous polyposis syndromes with high risk of colorectal cancer include the autosomal dominant Familial Adenomatous Polyposis (FAP [MIM: 175100]); and the autosomal recessive *MUTYH* associated polyposis (MAP [MIM: 608456]) syndrome. Recently two new entities have been described – the autosomal recessive *NTHL1* associated polyposis (NAP [MIM: 616415])^2^ and the autosomal dominant polymerase proofreading-associated polyposis (PPAP) syndrome due to mutations in *POLD1* [MIM: 174761] and *POLE* [MIM: 174762] genes^3, 4^.

FAP is characterized by the early onset of hundreds to thousands of adenomatous polyps throughout the colon and rectum with over 90% risk of development of carcinoma in one or more of the polyps^1^. FAP is caused by germline mutation in the *APC* gene^5^. *APC* is an integral part of the wnt-signalling mechanism and regulates the proliferation of colonic epithelial cells^6^. *APC* mutation carriers also have an increased risk of developing small bowel, upper gastrointestinal and papillary thyroid carcinoma as well as childhood medulloblastoma and hepatoblastoma^7^. Benign manifestations like congenital hypertrophy of the retinal pigment epithelium (CHRPE), desmoid tumors, osteomas and dental anomalies are also common^7^. Correlation between the location of mutations in *APC* gene (genotype) and the clinical phenotype in terms of the number of polyps, age of onset of polyps and CRC and distinct extracolonic manifestations is well described^8^. An attenuated variant of FAP (AFAP) due to mutations in 5′ or 3′ end of the *APC* gene, is characterized by polyps not exceeding 100 and late age of onset^1, 5^. Up to 10% of FAP cases in whom *APC* mutation is not identified, there is bi-allelic germline mutation in the *MUTYH* gene^5^. Unlike FAP, the *MUTYH* associated polyposis has a lower polyp burden which rarely exceeds 100^5, 9^. Comprehensive genetic analysis of *APC* and *MUTYH* fails to identify underlying gene mutation in 10–20% of FAP cases^2, 4, 10, 11^ and only a small proportion of these are explained by the recently described NAP and PPAP syndromes^2, 4^.

Current knowledge regarding the spectrum of *APC* gene mutation, mutational hotspots and the genotype phenotype correlations is derived mainly from studies in Caucasian cohorts^5, 8, 12^. In recent years, studies from other geo-ethnic groups have identified several novel *APC* genotypes, phenotypes and genotype-phenotype associations^10, 13–16^. The underlying reason for differences in phenotypic associations has not been investigated but may be due to difference in the underlying genetic background or dietary habits^17, 18^. *APC* genotype-phenotype association studies in different geo-ethnic groups can enrich the existing knowledge about phenotypic consequences of distinct *APC* mutations and guide counseling and risk management in different populations. This is the first FAP cohort being reported from South Asia and the only non-Caucasian cohort with comprehensive molecular genetic analysis of all the five adenomatous polyposis associated genes (*APC*, *MUTYH*, *NTHL1*, *POLD1* and *POLE*).

## Results

The 53 unrelated Indian FAP families reported here represent the diverse regions and religions of the Indian subcontinent with 15 hailing from northern, 15 from eastern, 14 from western and 9 from southern states of India and belonging to Hindu (46), Muslim (2), Christian (3) and Jain (2) religions. Of the 53 probands, 25 had no family history of polyposis or cancer suggesting a *de novo* mutation. The remaining 28 probands reported a family history of polyposis with or without CRC or other extracolonic manifestations. All the probands had classical polyposis except three AFAP cases with <100 adenomatous polyps. Through Sanger sequencing and MLPA of *APC* and *MUTYH* genes, 45 families were found to harbor deleterious germline mutation in the *APC* gene (35 distinct mutations) and 2 families with bi-allelic *MUTYH* gene mutation. With extended testing of family members, a total of 60 carriers of *APC* mutation and 4 carriers of bi-allelic *MUTYH* mutations were identified. In a combined analysis in 60 *APC* mutation carriers and their 58 untested relatives with FAP associated cancer or benign manifestation, the phenotypic features observed were 79 CRC, 5 upper GI cancers, 3 thyroid cancer, 2 brain tumors, 13 desmoid tumors/fibromatosis. CHRPE was noted in 14/34 *APC* mutation carriers for whom fundus examination details were available.

### Mutation spectrum

**Figure 1:**
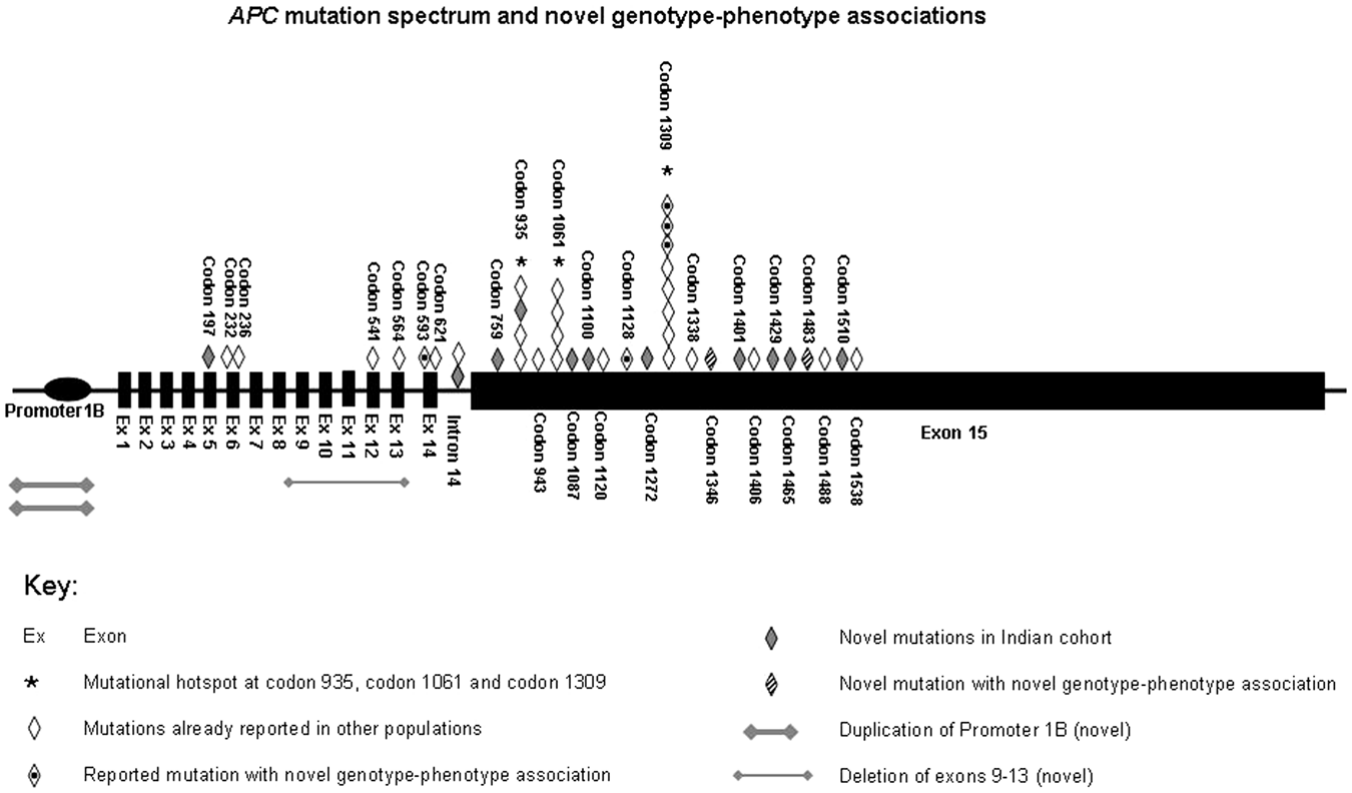
*APC* mutation spectrum and novel genotype-phenotype associations. The mutation distribution shows clustering of two thirds of all *APC* mutations in proximal Exon 15, with three Indian mutational hotspots (codon 935, 1061 and 1309) contributing to one third of all *APC* mutations. Large number of novel *APC* mutations (n = 15) and few novel genotype phenotype associations for codon 1228, 1346 and 1483 mutations.

Of the 35 distinct *APC* mutations described in Table 1 and Fig. 1, 15 (43%) were *novel* mutations not previously described in the literature or the InSiGHT database. Vast majority of the mutations were truncating (17 frameshift & 14 nonsense), 2 splice site and 2 large genomic rearrangements (LGR). All the mutations were between codons 197 to 1538. The proximal exon 15 harbored 24 (69%) of all the mutations. A 5 base pair deletion at codon 1309 (c.3927_3931delAAAGA) was the most frequent mutation, identified in 7 unrelated families. A 2 base pair deletion affecting the same codon 1309 (c.3925_3927delGA) was identified in an additional family. Codon 1061 mutation (c.3183_3187delACAAA) was identified in 4 families. Interestingly, 4 distinct truncating mutations at codon 935 occurred due to 4 different nucleotide alterations (c.2804dupA, c.2805_2815del11, c.2805 C > A and c.2802_2805delTTAC) in 4 families. The remaining 28 mutations were rare and identified in one family each. The *APC* LGRs identified were a duplication of the Promoter1B identified in two families and deletion of exons 9–13 in one family. In 2 of the 3 AFAP cases, biallelic *MUTYH* mutations were identified. A homozygous *MUTYH* mutation E466X (now E480X) was identified in a South Indian Tamil AFAP patient with 40 polyps and CRC. Compound heterozygous *MUTYH* mutations R241W and G286E were identified in a case with less than 100 polyps. In the 6 *APC* and *MUTYH* mutation negative cases with classical FAP phenotype, sequencing of the entire coding region of *NTHL1* gene and the exonuclease domain of *POLD1* gene (exons 6–13) and *POLE* gene (exons 9–14) did not identify any mutation.

**Table 1:** Spectrum of *APC* mutations in Indian FAP cohort.

Sr. No	Nucleotide change	Exon	Consequence	Type of mutation	No. of families with this mutation	Reported in InSiGHT^*^ database or novel
1	c.589delA	5	p.R197Efs*8	Frameshift	1	Novel
2	c.706 C > T	6	p.Q236*	Nonsense	1	Reported
3	c.694 C > T	6	p.R232*	Nonsense	1	Reported
4	c.1620dupA	12	p.Q541Tfs*19	Frameshift	1	Reported
5	c.1690C > T	13	p.R564*	Nonsense	1	Reported
6	c.1779G > A	14	p.W593*	Nonsense	1	Reported
7	c.1861dupA	14	p.T621Nfs*13	Frameshift	1	Reported
8	c.2274delA	15	p.A759Pfs*2	Frameshift	1	Novel
9	c.2802_2805delTTAC	15	p.Y935Ifs*19	Frameshift	1	Reported
10	c.2804dupA	15	p.Y935*	Frameshift	1	Reported
11	c.2805_2815del11	15	p.Y935*	Frameshift	1	Novel
12	c.2805 C > A	15	p.Y935*	Nonsense	1	Reported
13	c.2828 C > G	15	p.S943*	Nonsense	1	Reported
14	c.3183_3187del5	15	p.Q1062*	Frameshift	4	Reported
15	3259_3260delCT	15	p.L1087Qfs*31	Frameshift	1	Novel
16	c.3298dupT	15	p.S1100Ffs*19	Frameshift	1	Novel
17	c.3358 G > T	15	p.G1120*	Nonsense	1	Reported
18	c.3682 C > T	15	p.Q1228*	Nonsense	1	Reported
19	c.3815 C > A	15	p.S1272*	Nonsense	1	Novel
20	c.3925-3926delGA	15	p.E1309Lfs*5	Frameshift	1	Reported
21	c.3927_3931del5	15	p.E1309Dfs*4	Frameshift	7	Reported
22	c.4012 C > T	15	p.Q1338*	Nonsense	1	Reported
23	c.4037 C > G	15	p.S1346*	Nonsense	1	Novel
24	c.4202_4203delTT	15	p.I1401Sfs*7	Frameshift	1	Novel
25	c.4216 C > T	15	p.Q1406*	Nonsense	1	Reported
26	c.4285 C > T	15	p.Q1429*	Nonsense	1	Novel
27	c.4387_4394dup8	15	p. S1465Rfs*11	Frameshift	1	Novel
28	4446delT	15	p.P1483Qfs*24	Frameshift	1	Novel
29	c.4463 T > G	15	p.L1488*	Nonsense	1	Reported
30	c.4529delG	15	p.S1510Tfs*13	Frameshift	1	Novel
31	c.4612_4613delGA	15	p.E1538Ifs*5	Frameshift	1	Reported
32	IVS14 + 1 G > A	—	—	Splice site	1	Reported
33	IVS14 + 2 T > C	—	—	Splice site	1	Novel
34	Deletion of Exons 9-13	—	—	LGR	1	Novel
35	Duplication of promoter 1B	—	—	LGR	2	Novel

### Phenotypic features and rare genotype-phenotype associations

Of the 60 *APC* mutation carriers, 31 had developed CRC at a mean age of 38.3 years (range18–53 years) in a background of classical polyposis with hundreds to thousands of polyps in all but one case of AFAP with only 50 polyps. In 23 *APC* carriers, polyposis was diagnosed at a mean age of 32 years (range: 9–60 years) without CRC on endoscopic evaluation or histopathological examination of prophylactic procto-colectomy. In the remaining 6 carriers, colonoscopy was yet to be performed or its details were not available. Six *APC* carriers developed extracolonic cancers with or without CRC. These included 2 cases with papillary thyroid cancer, 1 case with duodenal cancer, 1 case with intracranial germinoma, 1 case with papillary thyroid carcinoma and duodenal cancer, and 1 case with duodenal cancer and small intestine cancer. One or more benign extracolonic manifestations were identified in 27/60 *APC* mutation carriers. These included CHRPE (n = 14), desmoid tumor or fibromatosis (n = 13), upper GI polyps (n = 8) and osteomas (n = 3). Eight very rare FAP phenotypes or phenotypes rarely associated with mutations outside specific regions of the *APC* gene were observed. These include the second reported case of intracranial germ cell tumor in an *APC* carrier^19^, absence of profuse polyposis and early onset CRC in 3 of the 7 codon 1309 mutation carriers as is classically described^20^, attenuated phenotype with only 50 polyps at age 33 years in a codon 593 mutation carrier, desmoid tumor with codon 1228 mutation, papillary thyroid cancer with codon 1346 mutation and most interestingly CHRPE with codon 1483 mutation^7, 8^.

## Discussion

In FAP, the mutation spectrum of *APC* gene and genotype-phenotype correlations is well characterized for the Caucasian population^12, 21–25^, and to some extent for the East Asian population^15, 26–30^. Also, comprehensive molecular characterization of all the 5 known genes has been performed in very limited number of cases, that too only in the Caucasian population. Our study is the first report of a South Asian cohort of 53 FAP families and the only non-Caucasian FAP cohort analysed for all the 5 adenomatous polyposis associated genes.

The wide variation in the reported frequency of germline *APC* or *MUTYH* mutations in FAP cohorts from as low as 40–60%^23, 31, 32^ to as high as 75–94%^10, 24, 33^ is due to the stringency in making a syndromic diagnosis or lack of comprehensive genetic analysis. The high mutation detection rate of 89% in our cohort reflects the appropriateness of our clinical characterization for making the syndromic diagnosis and the comprehensive genetic analysis for *APC* and *MUTYH* including MLPA.

This study has identified a new Indian mutational hotspot at codon 935 seen in 4 (9%) FAP families. In addition, the two other known hotspot mutations at codons 1309 and 1061 were seen in 18% and 9% families respectively. High frequency of codon 1309 and 1061 mutations worldwide^32^ is a result of repetitive nucleotides in DNA sequence making it a mutational hotspot. Identification of *APC* LGR in 3 of the 11 families negative for *APC* point mutation or small indels and biallelic *MUTYH* mutation in 2 of the 8 families without *APC* mutation or LGR mandates its inclusion in comprehensive genetic analysis for south Asian FAP/AFAP cases. The *MUTYH* mutation E466X (now E480X), previously described in 3 unrelated Indian families living in the UK^34^ was identified as a homozygous mutation in one of our AFAP case from Tamil Nadu in south India. E466X may thus be a founder *MUTYH* mutation in Indians, possibly of Tamil ancestry. The founder effect of E466X needs to be confirmed with haplotyping studies and its population frequency can be established in a larger cohort. *NTHL1*, *POLD1* or *POLE* mutations were not identified in any of the 6 FAP probands negative for *APC* or *MUTYH* mutations. This is not surprising as none of these families fulfilled the salient features of PPAP or NAP as described in the literature^4, 35^.

Of the 35 distinct mutation identified in our cohort, 15 (43%) are novel and not previously reported in Caucasian or other geo-ethnic groups. Moreover eight very rare FAP phenotype or phenotypes rarely associated with mutations outside specific regions of the *APC* gene were identified. *APC* genotypes and genotype-phenotype associations rarely or never observed in Caucasian cohorts are being increasingly reported from other geo-ethnic groups^10, 14–16, 29^. This highlights the need to study different geo-ethnic groups to enrich the global *APC* mutational spectrum and expand our knowledge of phenotypic associations of distinct *APC* mutations.

**Figure 2:**
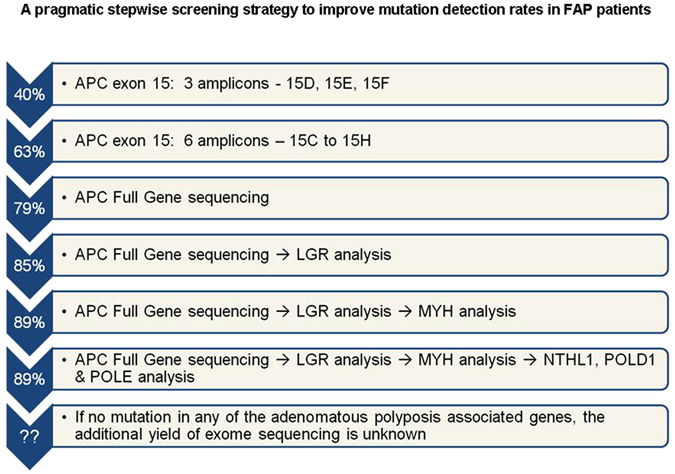
A pragmatic stepwise screening strategy to improve mutation detection rates in FAP patients. Cumulative mutation detection rates with step wise screening of exons/genes most likely to be mutated in south Asian FAP cases. Arrows on left side shows the cumulative mutation detection rates in our cohort achieved after each step. In our cohort, the cumulative mutation detection rate did not change with *NTHL1*, *POLD1* and *POLE* gene analysis it may increase the detection rate slightly in larger cohorts of *APC* and *MUTYH* negative adenomatous polyposis cases from different geo-ethnic background.

Based on the mutational spectrum and hotspots identified, a pragmatic stepwise genetic testing algorithm is proposed for FAP cases in south Asian countries where genetic testing is not routinely performed due to resource constraints (Fig. 2). Initial screening of three amplicons (15D–15F) harboring the mutational hotspot codons 1309, 1061 and 935 could identify 40% of all *APC* mutations and sequencing of additional 3 amplicons of exon 15 (15 C, 15 G, 15 H) could identify two thirds of all *APC* mutations. If no mutation is identified rest of the *APC* should be screened followed by LGR analysis and *MUTYH* gene sequencing. Extended testing of other adenomatous polyposis associated genes (*NTHL1*, *POLD1 and POLE)* may be considered but the yield is likely to be very low. The present study and few recent reports^36^ highlight that a significant proportion of FAP cases do not harbor pathogenic mutations in the genes known to be associated with FAP, MAP, NAP, PPAP syndrome. Germline exome sequencing in an adenomatous polyposis cohort has recently reported loss-of-function germline mutations in a few promising candidate genes (*DSC2*, *PIEZO1*, *ZSWIM7*)^36^ and biallelic mutations in *MSH3* gene^37^. However these recently identified adenomatous polyposis genes are likely to remain under-reported, unless they are tested as single genes or included in multi-gene next generation sequencing (NGS) panels. The currently used multi-gene panels may not be informative as they do not include *NTHL1*, *POLD1* and *POLE* genes. Therefore there is a need to conduct comprehensive genetic analysis of all the known adenomatous polyposis genes or exome sequencing studies in large pooled cohorts of *APC* and *MUTYH* negative adenomatous polyposis cases with detailed phenotypic and geo-ethnicity correlation.

In conclusion, the comprehensive investigation of all the five adenomatous polyposis genes in a well characterized Indian FAP cohort confirms the high frequency of *APC* mutations in classical FAP, *MUTYH* in AFAP cases and absence of *NTHL1*, *POLD1* and *POLE* mutations in cases not showing syndromic features of PPAP or NAP. The pragmatic stepwise approach proposed can improve uptake of genetic testing for FAP in south Asian countries. Identification of a large number of novel *APC* mutations and genotype phenotype associations that are rare in the Caucasian population highlights the need for comprehensive phenotypic characterization and genetic analysis in large FAP cohorts from diverse geo-ethnic backgrounds.

## Methods

### Patients and Phenotype characterization

The study was conducted on 53 FAP families recruited through Cancer Genetics Clinic at Tata Memorial Centre, Mumbai and Christian Medical College, Vellore; India. The study was approved by the Hospital Ethics Committee of the Tata Memorial Hospital and all participating subjects provided written informed consent. All experiments were carried out in accordance with the approved guidelines and regulations. Syndromic diagnosis of FAP or AFAP was based on the number of adenomatous polyps in the colorectum with or without colorectal cancer. Further phenotypic characterization was done based on colonoscopy, esophago-gastro-duodenoscopy (EGD), computerized tomography of abdomen, thyroid ultrasound and ophthalmic examination. Detailed family history and medical records were taken from all the families reported in this study. Genetic testing was extended on first and second degree relatives if a deleterious germline mutation was identified in the proband. Blood sample was collected from 112 members from these 53 families.

### PCR and Sequencing

For germline mutation analysis the complete coding sequence of the *APC*, *MUTYH* and *NTHL1* genes and the exonuclease domain of *POLD1* gene (exons 6–13) and *POLE* gene (exons 9–14) were amplified by Polymerase Chain Reaction (PCR). Primer sequences and annealing temperatures for PCR used are given in the supplementary Tables S1–S4. PCR products were purified with ExoSAP-IT [USB products, Affymetrix] and sequenced using an ABI 310 Avant, 3500 and 3730 DNA sequencer (Applied Biosystems). All mutations were confirmed by bidirectional sequencing. For most of the cases, the mutations were further reconfirmed on a second independent sample collected after the identification of mutation. InSiGHT database (LOVD) and available literature was used to check if the mutations identified was reported or novel. The mutations identified in our cohort are submitted in the InSiGHT database (www.insight-group.org).

### MLPA analysis

If no *APC* mutation was identified on sequencing, large genomic rearrangement (LGR) in *APC* and *MUTYH* gene were evaluated with Multiplex ligation-dependent probe amplification (MLPA) using the SALSA MLPA *APC* P043 kit [MRC-Holland] as per the instructions provided by the company. The data was analyzed with Coffalyser software. All deletions or duplications identified and all uncertain results were confirmed in at least two independent MLPA reactions.

## Electronic supplementary material

Supplementary information
