# Supplementary material for: Novel mutations and phenotypic associations identified through APC, MUTYH, NTHL1, POLD1, POLE gene analysis in Indian Familial Adenomatous Polyposis cohort
Source: Sci Rep. 2017 May 22;7:2214. doi: 10.1038/s41598-017-02319-6 (PMC5440391; doi:10.1038/s41598-017-02319-6)
Supplement: Supplementary file 1 — Supplementary information [file 41598_2017_2319_MOESM1_ESM.pdf]

Supplementary Information

Novel mutations and phenotypic associations identified through *APC, MUTYH, NTHL1, POLD1, POLE* gene analysis in Indian Familial Adenomatous Polyposis cohort

Nikhat Khan[1, 2], Anuja Lipsa[1, 2], Gautham Arunachal[3], Mukta Ramadwar[2, 4], Rajiv Sarin[1, 2]*

1. Sarin Lab, Advanced Centre for Treatment, Research and Education in Cancer (ACTREC)-Tata Memorial Centre, Navi Mumbai, India
2. Homi Bhabha National Institute, Training School Complex, Anushakti Nagar, Mumbai 400085, India
3. Clinical Genetics Unit, Christian Medical College and Hospital, Vellore, India
4. Department of Pathology, Tata Memorial Hospital-Tata Memorial Centre, Mumbai, India

Table S1: *APC* primer sequences and Annealing temperatures

Exon	Primer sequences	Annealing temperature (°C)
APC 1F	TTTCTTTAAAAACAAGCAGCCA	59°C
APC 1R	CACAGAAAACCTTGCCTCAG	
APC 2F	AAGGTGCGTGCTTTGAGAGT	59°C
APC 2R	ACCAACACCCAAATCGAGAG	
APC 3F	CCAAGTGGACTTTTCAGGGA	59°C
APC 3R	CTGGAGTACACAAGGCAATGTT	
APC 4F	GCTCTTCTGCAGTCTTTATTAGCA	59°C
APC 4R	CCTAGTTGAACCCTGAGGTCC	
APC 5F	AAGCCACTTGTGACTTTGGC	62°C
APC 5R	GTTGCTCAGCAGCCATGATA	
APC 6F	TGCGGTGAGCTGAGATTATG	62°C
APC 6R	ACCCACAAACAAGAAAGGCA	
APC 7F	GCAGCTCTAATGCTCAAGGG	59°C
APC 7R	TGGTACTGAATGCTTCTGGAAA	
APC 8F	CCATTCTGCAGTTTAATGCTCA	59°C
APC 8R	TAGAGATGGGGTTTTGCCAC	
APC 9F	CTGGAAAGGTTTTCCGGTTT	59°C
APC 9R	TGCTTTGAAACATGCACTACG	
APC 10F	GTCAAGGGCAGATGAGTGGT	65.6°C
APC 10R	TTCTATGCTGGAAACCAGGG	
APC 11F	TTGTCTTTTTCCTCTTGCCC	59°C
APC 11R	AGCGAATGTGAAGCACAGGT	
APC 12F	CCTGTTGCTTATCATTTCTCACC	64.2°C
APC 12R	AGAGTGAGACCCTGCCTCAA	
APC 13F	CAGCCTCCCAAAGTGATAGG	50.2°C
APC 13R	ATGGCTAAAAGAAGGCAGCA	
APC 14F	AGGGACGGGCAATAGGATAG	59°C
APC 14R	CATTGCTTACAATTAGGTCTTTTTGA	
APC 15AF	AGAGTGGCACCCAACCATAG	59°C
APC 15AR	TCCCATAATGCTTCCTGGTC	
APC 15BF	GTTACTGCATACACATTGTGAC	55°C
APC 15BR	GCTTTTTGTTTCCTAACATGAAG	
APC 15CF	GCTCAAGCTTGCCATCTCTT	62°C
APC 15CR	TATGGGCAGCAGAGCTTCTT	
APC 15DF	CCAGGAACTTCTTCAAAGCG	62°C
APC 15DR	GTGAAGGACTTTGCCTTCCA	
APC 15EF	GTCAATACCCAGCCGACCTA	59°C
APC 15ER	AGGCTGATCCACATGACGTT	
APC 15FF	AACGTCATGTGGATCAGCCT	62°C
APC 15FR	TGCTGGATTTGGTTCTAGGG	

APC 15GF	CAGACGACACAGGAAGCAGA	62°C
APC 15GR	GCAGCTTGCTTAGGTCCACT	
APC 15HF	GTGAACCATGCAGTGGAATG	59°C
APC 15HR	TGTTGGCATGGCAGAAATAA	
APC 15IF	TTTGCCACGGAAAGTACTCC	59°C
APC 15IR	TATCATCCCCCGGTGTAAAA	
APC 15JF	CCCAGACTGCTTCAAAATTACC	59°C
APC 15JR	GAGCCTCATCTGTACTTCTGC	
APC 15KF	CCCTCCAAATGAGTTAGCTGC	59°C
APC 15KR	TTGTGGTATAGGTTTTACTGGTG	
APC 15LF	ACCCAACAAAAATCAGTTAGATG	59°C
APC 15LR	GTGGCTGGTAACTTTAGCCTC	
APC 15MF	ATGATGTTGACCTTTCCAGGG	59°C
APC 15MR	ATTGTGTAACTTTTCATCAGTTGC	
APC 15NF	AAAGACATACCAGACAGAGGG	59°C
APC 15NR	CTTTTTTGGCATTGCGGAGCT	
APC 15OF	AAGATGACCTGTTGCAGGAATG	59°C
APC 15OR	GAATCAGACGAAGCTTGTCTAGAT	
APC 15PF	CCATAGTAAGTAGTTTACATCAAG	55°C
APC 15PR	AAACAGGACTTGTACTGTAGGA	
APC 15QF	CAGCCCCTTCAAGCAAACATG	59°C
APC 15QR	GAGGACTTATTCCATTTCTACC	
APC 15RF	CAGTCTCCTGGCCGAAACTC	62°C
APC 15RR	GTTGACTGGCGTACTAATACAG	
APC 15SF	TGGTAATGGAGCCAATAAAAAGG	59°C
APC 15SR	TGGGAGTTTTCGCCATCCAC	
APC 15TF	TGTCTCTATCCACACATTCGTC	59°C
APC 15TR	ATGTTTTTCATCCTCACTTTTTGC	
APC 15UF	GGAGAAGAACTGGAAGTTCATC	59°C
APC 15UR	TTGAATCTTTAATGTTTGGATTTGC	
APC 15VF	TCTCCCACAGGTAATACTCCC	59°C
APC 15VR	GCTAGAACTGAATGGGGTACG	
APC 15WF	CAGGACAAAATAATCCTGTCCC	53°C
APC 15WR	ATTTTCTTAGTTCTATTCTTCCTC	

Table S2: *MUTYH* primer sequences and annealing temperatures

Exons	Primer sequences	Annealing temperature (ºC)
MUTYH_2F	AAAATTTGGCCTCATTGTGAC	60-53ºC Touchdown PCR
MUTYH_2R	TATCACAATCCCTTCCCAGC	
MUTYH_3+4+5F	AAGGGGGTTAGTTGGGGGAAGC	60-53ºC Touchdown PCR
MUTYH_3+4+5R	CAAGGGTGAAGGTGGTAGAGGAAGC	
MUTYH_6+7+8F	TTTGGGGTGGGTGTAGAGAAGG	60-53ºC Touchdown PCR
MUTYH_6+7+8R	GCACAGAGGGGCCAAAGAGTTAG	
MUTYH_9+10+11F	CAGCCCACCCCCACTTTGT	60-53ºC Touchdown PCR
MUTYH_9+10+11R	GCTTTGGCCGGGTTCTGC	
MUTYH_12+13F	TCTAGGTTGGCCCCTAAAGC	60-53ºC Touchdown PCR
MUTYH_12+13R	GTCAAGGGGTTCAAATAGGC	
MUTYH_14F	TTGGCTTTTGAGGCTATATCC	60-53ºC Touchdown PCR
MUTYH_14R	ACATGTAGGAAACACAAGGAAGTA	
MUTYH_15F	TGGGACATGAAGTTAAGGGC	60-53ºC Touchdown PCR
MUTYH_15R	GAGTGGAGAATGTTCACCCAG	
MUTYH_16F	GAGAGGATTCTCTGCTCCCC	60-53ºC Touchdown PCR
MUTYH_16R	TCGAAACCAGTCTGAGCAAC	

Table S3: *POLD1* and *POLE* primer sequences** and annealing temperatures

Gene_Exon	Primer sequences	Annealing temperatures (ºC)
POLE_Exon 9F	AACCAGAGGGAGGTAGAGCA	63.2 ºC
POLE_Exon 9R	CTCCCTGTTGGTGATGAGGT	
POLE_Exon 10+11F	GCACTTTCACATTGCTGTGG	63.2 ºC
POLE_Exon 10+11R	CCTAAGTCGACATGGGAAGC	
POLE_Exon 12F	ACACGTCCAGGAGACCAAAC	63.2 ºC
POLE_Exon 12R	TTGCAGCTGCCATACTCTTG	
POLE_Exon 13F	GGTGCCTGTTAGGAACTTGC	61.4 ºC
POLE_Exon13R	GAGCGGGCTGGCATACAT	
POLE_Exon 14F	GGCTTTGCTTTCTGTGCTTC	63.2 ºC
POLE_Exon 14R	AGCACTCCTGGGACATCCAC	
POLD1_Exon 6+7+8F	CTCCCGAGAGAGTGAGTGCT	63.2 ºC
POLD1_Exon 6+7+8R	ATCTCCACACCCTCTGTGCT	
POLD1_Exon 9F	AGGTGAGAGCAGAGCAGGAG	63.2 ºC
POLD1_Exon 9R	AGGAGCTGATGGCTCAGGAC	
POLD1_Exon 10F	GGTTCTGCAGGATTTTCAGG	63.2 ºC
POLD1_Exon 10R	GTGGAGAGGGAGTGGGAAG	
POLD1_Exon 11+12F	TGTCCCCAAATCTCTTCCTG	61.4 ºC
POLD1_Exon 11+12R	CCAGGAAAGCAGAGACAAGG	
POLD1_Exon 13F	TCCCTGACCCCATCCGTG	63.2 ºC
POLD1_Exon 13R	GGACAAGTCTCGGCTACTGA	

** Taken from Bellido, F. et al. (Reference No. 3 in manuscript)

Table S4: *NTHL1* primer sequences and annealing temperatures

**Exons**	**Primer sequences**	**Annealing temperature (ºC)**
NTHL1_1F	GGCCGCATGGGCCGCCGGG	63ºC
NTHL1_1R	AGCCTGGAGTGGAGAGTCC	
NTHL1_2F	GTCGCTGGCATAAGGAGG	64.2ºC
NTHL1_2R	CGAGCACGAGGCCCTAAACC	
NTHL1_3F	AAGTGCTGGGATGACGGGTG	64.2ºC
NTHL1_3R	GCCTGAGATGCTTGACCCT	
NTHL1_4F	GAGCCTACACGTGCATCATTG	64.2ºC
NTHL1_4R	GGTGCTCAGCCCATGTGAC	
NTHL1_5+6F	GATGGTCACATGTAGGCTTGC	57ºC
NTHL1_5+6R	CCATCTGCAAACACACCAAAG	
